# Impact of COVID-19 pandemic on the utilization of routine immunization services in Lebanon

**DOI:** 10.1371/journal.pone.0246951

**Published:** 2021-02-17

**Authors:** Ziad Mansour, Jinan Arab, Racha Said, Alissar Rady, Randa Hamadeh, Bernard Gerbaka, Abdul Rahman Bizri

**Affiliations:** 1 Connecting Research to Development, Beirut, Lebanon; 2 World Health Organization Lebanon Country Office, Beirut, Lebanon; 3 Ministry of Public Health, Beirut, Lebanon; 4 Division of Pediatric Intensive Care Unit and Pediatric Emergencies, Department of Pediatrics, Hôtel-Dieu de France, Beirut, Lebanon; 5 Division of Infectious Diseases, Department of Internal Medicine, American University of Beirut Medical Center, Beirut, Lebanon; University of New South Wales, AUSTRALIA

## Abstract

**Introduction:**

The global abrupt progression of the COVID-19 pandemic may disrupt critical life-saving services such as routine immunization (RI), thus increasing the susceptibility of countries to outbreaks of vaccine-preventable diseases (VPDs). Being endemic to several infectious diseases, Lebanon might be at increased risk of outbreaks as the utilization of RI services might have deteriorated due to the pandemic and the country’s political unrest following the October 2019 uprising. The aim of this study was to assess the changes in the utilization of RI services in both the public and private sectors following the COVID-19 pandemic.

**Methods:**

A self-administered cross-sectional survey was completed electronically, in April 2020, by 345 private pediatricians who are registered in professional associations of physicians in Lebanon and provide immunization services at their clinics. Means of the reported percentages of decrease in the utilization of vaccination services by pediatricians were calculated. As for the public sector, an examination of the monthly differences in the number of administered vaccine doses in addition to their respective percentages of change was performed. Adjustment for the distribution of RI services between the sectors was performed to calculate the national decrease rate.

**Results:**

The utilization of vaccination services at the national level decreased by 31%. In the private sector, immunization services provision diminished by 46.9% mainly between February and April 2020. The highest decrease rates were observed for oral poliovirus vaccine (OPV) and hepatitis A, followed by measles and pneumococcal conjugate vaccines. The number of vaccine doses administered in the public sector decreased by 20%. The most prominent reductions were detected for the OPV and measles vaccines, and during October 2019 and March 2020.

**Conclusion:**

The substantial decrease in the utilization of RI as a result of the COVID-19 pandemic requires public health interventions to prevent future outbreaks of VPDs.

## Introduction

The global abrupt progression of the COVID-19 pandemic may increase the risk of disruption of critical life-saving services such as routine immunization (RI) due to the diversion of the resources and efforts of healthcare systems towards supporting the response to the pandemic, in addition to the alterations in health-seeking behaviors as a result of social distancing requirements and public hesitancy. Interruption of vaccination services will increase the susceptibility of countries to outbreaks of vaccine-preventable diseases (VPDs) including measles and polio among others, as large pockets of unvaccinated infants and young children emerge [1]. The occurrence of such outbreaks will jeopardize the health status of the most vulnerable and will impose an additional burden on health systems already drained by the COVID-19 pandemic [2].

COVID-19 is the not the first infectious disease to be tackled while facing challenges to prevent outbreaks of others [3]. In 2019, an unprecedented measles outbreak, attacking 341,000 people, emerged in the shadow of the Ebola epidemic in the Democratic Republic of Congo (DRC) and killed twice as much as Ebola [4]. Similarly, in 2015, Guinea suffered from a measles outbreak post-Ebola [5]. Sharp drops in measles vaccination coverage, because of healthcare systems battered by Ebola, were mainly behind the resurgence of the disease [6].

Experience from prior outbreaks occurring following considerable deteriorations in immunization rates has highlighted the urgency of maintaining RI services. For instance, in Samoa, 83 deaths within 3 months were attributed to a measles outbreak following a 50% drop in vaccination coverage [7]. Likewise, Ukraine has been witnessing a series of outbreaks of VPDs ever since its vaccination rates started decreasing tremendously over the years [8, 9]. Multiple measles outbreaks with more than 12,000 cases have occurred and polio resurged for the first time in two decades, making Ukraine the sole country not declared as polio-free in Europe [10, 11].

Recognizing the disastrous consequences of the decline in immunization rates, The World Health Organization (WHO) has issued, on March 26^th^, 2020, interim guidance for immunization services during the COVID-19 pandemic. The guidance recommended sustainment of RI programs while preserving the safety of health workers and populations, temporary suspension of mass immunization campaigns based on individual risk-benefit assessments, and the establishment of innovative strategies to assess immunity gaps and track unvaccinated children [12].

### In Lebanon

The government announced the state of General Mobilization on March 15^th^. This entailed many public health and social measures such as physical distancing of at least 2 meters apart (however, adapting to it has been difficult), a curfew, closure of businesses in specific sectors and of education institutions, alternate car circulation policy, and closure of points of entry. Being endemic to several VPDs, Lebanon has aligned itself with WHO guidance on immunization during the COVID-19 pandemic and with the priorities of the Expanded Program on Immunization (EPI). The Lebanese national health authorities recommended the sustainment of immunization services’ provision in primary healthcare centers and private clinics while adopting an appointment system and ensuring the adherence to infection prevention and control measures (IPC) (social distancing measures, correct use of personal protective equipment, screening for COVID-19, hand hygiene and waste disposal) [13]. Yet, the complexity of this pandemic’s response with respect to obligatory confinement measures, the economic crisis ongoing in the country and the nationwide political unrest due to the October 17^th^, 2019 uprising may have potentially hindered the access to RI services and subsequently set the floor for the occurrence of outbreaks in the near future. As a further matter, the country has been attempting to recover from two consecutive measles outbreaks in 2018 and 2019 with more than 2,014 infected cases by implementing a national measles/polio campaign; however, the campaign has been temporarily postponed due to the pandemic, hence delaying the vaccination of around 600,000 children and further increasing the country’s susceptibility to measles outbreaks [14]. It’s worth noting that, based on the 2019 WHO-UNICEF coverage estimates data for Lebanon, the complete immunization coverage were all below the targeted 95%: oral polio virus 3 (OPV) (81%); diphtheria, tetanus, pertussis 3 (DTP) (83%); hepatitis B 3 (HepB) (80%); *haemophilus influenzae* type b 3 (Hib) (85%); and measles-containing vaccine (MCV) (63%) [15].

This research study was conducted to assess the changes in the utilization of RI services in both the public and private sectors as a result of the COVID-19 pandemic to project the occurrence of potential outbreaks of VPDs after the subsidence of the pandemic.

## Materials and methods

A combination of a cross-sectional survey completed by private pediatricians in April 2020 and an examination of official immunization data from the public sector were adopted in this study to assess the changes in the utilization of RI services between October 2019 and April 2020 as compared to the same period in the preceding year. Vaccination coverage is the traditional metric used to assess vaccine usage; however, the assessment of doses administered represents an immediately available proxy measure of coverage.

### Immunization in the public sector

To evaluate the changes in the utilization of RI services in the public sector, an examination and analysis of immunization data provided by the Ministry of Public Health were performed. The existing data included monthly reports of specific vaccine doses administered in primary healthcare centers and dispensaries for children under 1 year and covering the period between October 2019 and April 2020, as well as data from the previous year covering the same period. The reported vaccines are part of the national calendar for vaccination and included measles, OPV, pentavalent vaccine, inactivated poliovirus vaccine (IPV) and pneumococcal conjugate vaccine (PCV13) [16].

The monthly differences in the number of administered doses for each vaccine between the two periods were calculated in addition to their respective percentages of change.

### Cross-sectional survey among pediatricians in the private sector

#### Sampling

The study sample included pediatricians registered with any of the two professional associations of physicians in Lebanon [the Lebanese Order of Physicians (Beirut) and the Order of Physicians in North Lebanon (Tripoli)], practicing medicine in Lebanon and providing RI services. Given the likelihood of low response rates, an exhaustive sampling design was adopted in this study. The availability of practicing physicians’ databases from the two orders of physicians including their detailed contact information enabled the deployment of this sampling design. Physicians with incorrect or unavailable contact information, or with duplicated membership were excluded.

#### Data collection

A survey questionnaire was developed by Connecting Research to Development (CRD) public health team and subsequently reviewed and approved by national experts (S1 File). The questionnaire assessed the decrease in the overall utilization of RI services among private pediatricians during the period between October 2019 and April 2020 and in the provision of specific vaccines [OPV; inactivated poliovirus vaccine (IPV); measles; DTP; HepB; measles, mumps, rubella (MMR); PCV; and hepatitis A) from the national immunization calendar. Moreover, decrease rates in hepatitis A vaccine’s utilization were evaluated and served as an indicator reflecting the utilization of other vaccines that are not incorporated in the national public calendar, yet administered in the private sector as per the calendar developed by the Lebanese Pediatric Society. The tool was created and administered in English as it is a universal language among physicians in Lebanon. SurveyMonkey software was used to digitize the developed questionnaire for ease of accessibility and to decrease the survey burden on physicians. The questionnaire was self-administered and communicated via SMS text messages including a web-link to the survey. An SMS reminder text was sent in the week that followed to further enhance the response rate.

#### Data analysis

Surveys of pediatricians not providing RI services were excluded from the study. Descriptive statistics with proportions, and frequency distributions were conducted for categorical variables. Means of the reported percentages of decrease in the utilization of vaccination services by pediatricians were calculated. Data were analyzed using SPSS version 21.

#### Ethical considerations

Adherence to ethical guidelines was ensured throughout the research process. The study was approved by the Institutional Review Board (IRB) at Transforming Research to Development (TRD) as per reference number TRDIRB03020. Voluntary participation and withdrawal were granted as the survey questionnaire was self-administered online. The scope, aim and ethical considerations of the survey were rigorously explained in the introductory section and consent was obtained by answering the question “Do you agree to participate in this study?” Confidentiality was strictly applied during all study procedures. Surveys were anonymous and did not comprise any variable jeopardizing the identity of the participant or their patients.

## Results

### Private sector

Out of the 1,317 pediatricians who were invited to participate in the survey, 384 participants responded. However, the answers of 345 pediatricians, who reported providing RI services, were included in the analysis, yielding a response rate of 26.2%.

77.4% of physicians reported an overall decrease in the utilization of RI services between October 2019 and April 2020 as compared to the same period last year. Specifically, more than two-third of the respondents indicated a deterioration in the utilization rates of PCV vaccine (82.0%) and hepatitis A vaccine (79.8%) followed by DPT-containing vaccines (73.0%), measles/MMR vaccines (70.8%) and IPV vaccine (69.7%). The confinement period extending between February and April 2020 witnessed the most significant decrease rate in immunization services provision according to 87.6% of respondents (Table 1).

**Table 1:** Witnessed decrease among private physicians in the utilization of routine immunization services between October 2019 and April 2020.

	Number	Percentage
Physicians witnessing a decrease in the utilization of routine immunization services between October 2019 and April 2020	267	77.4
Physicians witnessing a decrease in the utilization of the following vaccines between October 2019 and April 2020 (N = 267)		
OPV	132	49.4
IPV	186	69.7
Measles	189	70.8
DPT	195	73.0
Hepatitis B	171	64.0
MMR	189	70.8
PCV	219	82.0
Hepatitis A	213	79.8
Period with the most significant decrease rate in immunization services provision in the private sector (N = 267)		
October- November 2019	6	2.2
December 2019- January 2020	21	7.9
February-April 2020	234	87.6
Do not know	6	2.2

On average, survey respondents estimated an overall decline in the utilization of RI by 46.9% in the private sector. Percent change reduction rates in the provision of specific vaccines ranged between 46% and 58% with the highest being reported for OPV (57.5%) and hepatitis A vaccines (57.2%) followed by PCV (53.3%), measles, (53.3%) and MMR vaccines (49.9%). The lowest percent decrease rate among the investigated vaccines was attributed to DPT-containing vaccines (46.3%) (Table 2).

**Table 2:** Percent decrease rate in the utilization of routine vaccination in the private sector between October 2019 and April 2020.

	Estimated Percent Decrease Rate
Average overall estimated percent decrease rate in the utilization of routine immunization services in the private sector between October 2019 and April 2020	46.9%
Average estimated percent decrease rates in the utilization of the following vaccines in the private sector between October 2019 and April 2020 (N = 267)	
OPV	57.5%
Hepatitis A	57.2%
Measles	53.3%
PCV	53.3%
MMR	49.9%
Hepatitis B	49.1%
IPV	48.5%
DTP	46.3%

### Public sector

**Fig 1:**
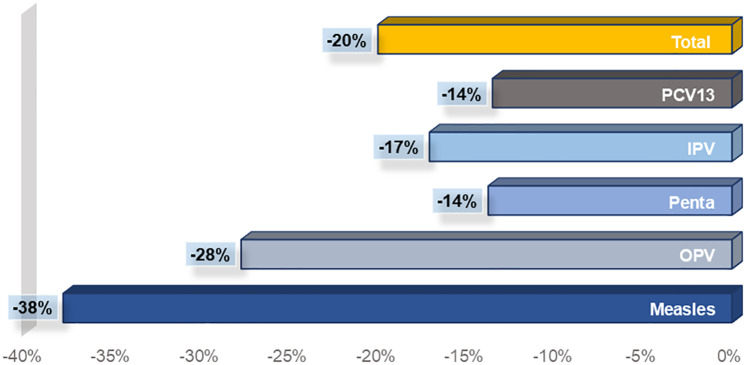
Overall changes in the number of vaccine doses administered in the public sector, between October 2019 and March 2020, as compared to the same period last year.

Analysis results of data from the public sector came in the same direction as those detected in the private sector. A negative difference of 71,567 doses was detected in the number of administered vaccines’ doses between October 2019 and March 2020 as compared to the same period last year, reflecting a 20% decrease in the utilization of routine vaccination services in the public sector. Among vaccines administered in the public sector, the highest negative percentage change was observed in measles vaccines (-38%) followed by OPV (-28%) (Fig 1).

**Fig 2:**
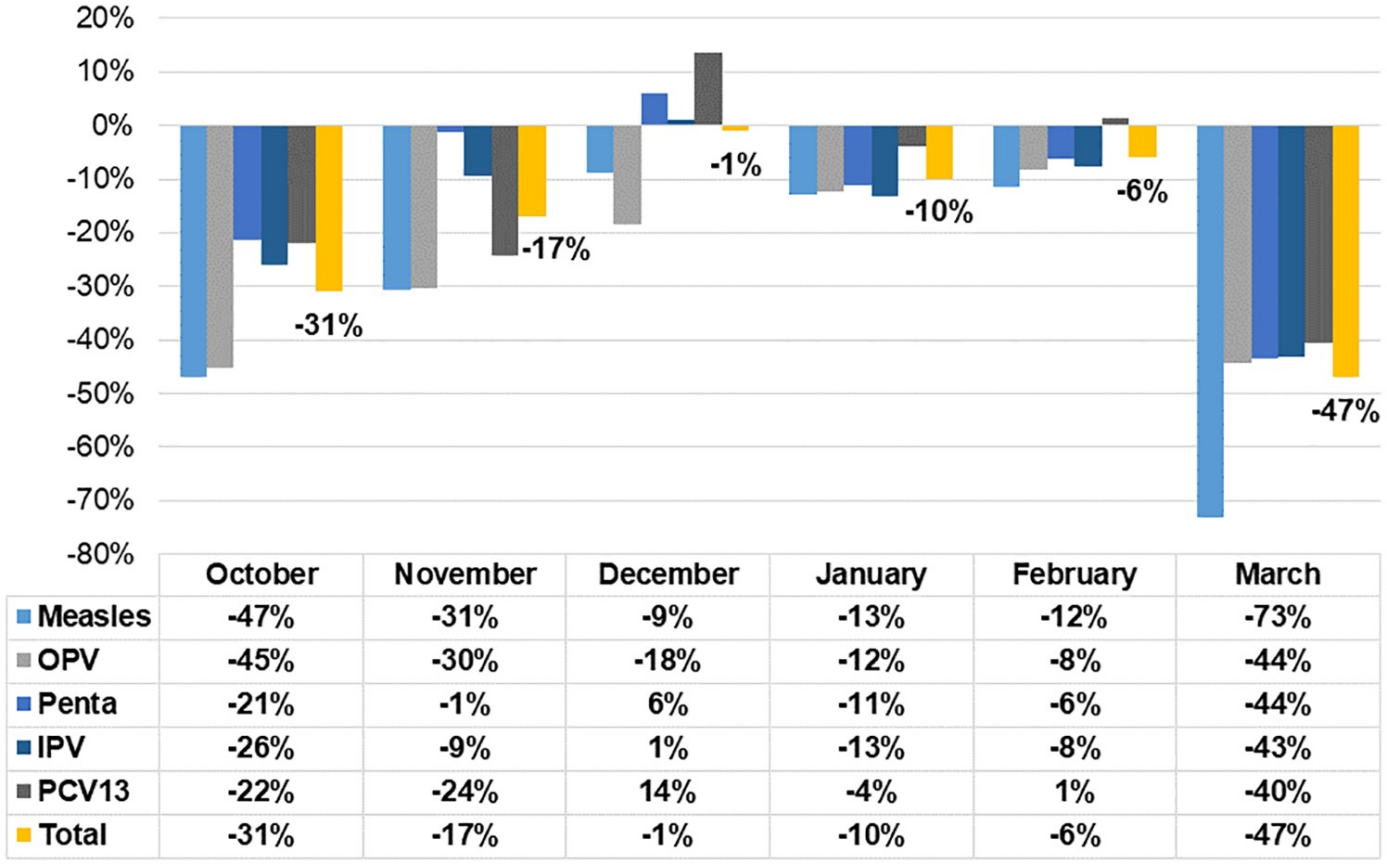
Monthly changes in the number of vaccine doses administered in the public sector as compared to the same period last year.

The examination of fluctuations in the monthly number of administered doses between the current and previous year revealed a negative difference throughout the study period. March witnessed the highest decrease in the total number of administered doses in the public sector (-47%) followed by October (-31%). In March, the substantial reduction was observed in measles vaccines whereby the number of administered doses declined by 73%. The decrease was slightly less prominent for OPV, Pentavalent, IPV and PCV and ranging from 40% to 44% (Fig 2).

### Utilization of routine immunization at the national level

Assessing the decrease in the utilization of RI services at the national level requires adjustment for the distribution of routine vaccination services between the public and private sectors. This was accomplished based on data from the Expanded Program on Immunization (EPI) Cluster survey indicating that around 60% of vaccination services are provided in the public sector and the rest in the private sector [17]. Adjustment results revealed a 31% decrease in the utilization of vaccination services at the national level.

## Discussion

This study assessed the changes in the utilization of RI services in both the public and private sectors in Lebanon between October 2019 and April 2020 as compared to the same period last year. Findings revealed a 31% decrease in the utilization of vaccination services at the national level. In the private sector, immunization services provision diminished to almost half with the greatest reduction being reported during the confinement period extending between February and April 2020. Utilization rates of all the investigated vaccines have dropped by almost half. The highest decrease rates were observed for OPV, hepatitis A, followed by measles and PCV-containing vaccines. The assessment of fluctuations in the number of vaccine doses administered in the public sector yielded a similar pattern, however with a lower decrease rate. The most prominent reductions were detected for the OPV and measles vaccines, and during October 2019 and March 2020.

To our knowledge, this study was the first to assess the impact of the COVID-19 pandemic on the utilization of vaccination services in Lebanon and the Middle East and North Africa (MENA) region. A similar study was conducted in England to assess the early impact of COVID-19 on routine childhood vaccination by April 2020 [18]. Additionally, another report with similar objectives entitled “Effects of the COVID-19 Pandemic on Routine Pediatric Vaccine Ordering and Administration—United States, 2020”, was published by Center for Disease Control and Prevention (CDC) on May 15^th^, 2020 and assessed the changes in the number of vaccine orders and the number of doses administered in the united states between January and April 2019 as proxy-indicators of vaccine usage. Report results were consistent with our study findings as noticeable reductions in the number of non-influenza vaccine doses, specifically measles-containing vaccines, were observed for children below 2 years of age. The report also highlighted the declaration of a national emergency as a start point for deterioration in vaccine usage. Similar outcomes were observed in our study as the highest decrease rates in the utilization of immunization services were witnessed after the declaration of the general mobilization plan during the month of March 2020 in the public sector, and during the confinement period extending between February and April 2020 for the private sector [19]. The substantial reduction in vaccination services observed in our study between October 2019 and January 2020 before the detection of the first coronavirus case in Lebanon can be explained by the state of civil unrest due to the October 17^th^, 2019 uprising coupled with road blockages and economic crisis. The road blockade were eased during December 2019 and thus private physicians regained their activities while the public sector was slower to recover as the trust of people in the State and all public services were deeply impacted by the news of corruption and inefficiency that accompanied the uprise.

As per our study findings, the utilization of immunization services has diminished by 31% at the national level between October 2019 and April 2020. The obtained results were comparable to those observed in previous epidemics such as Ebola. A study by Barden -O’Fallon et al. evaluating Ebola-related implications on newborn and child health service delivery and utilization in Guinea in both private and public facilities has revealed a 31% reduction in outpatient visits with child health services including immunization being the most affected by the Ebola epidemic [20]. Furthermore, our results indicated a 20% decrease in the provision of vaccination services at the primary healthcare level which was similarly observed in countries affected by the Ebola epidemic. A report entitled “Rapid assessment of Ebola impact on reproductive health services and service seeking behavior in Sierra Leone” indicated a 21% decrease in the proportion of children accessing primary healthcare units for key vaccination purposes during the Ebola epidemic [21]. Concerning the private sector, our findings highlighted a greater reduction in vaccine usage as compared to the public sector. An analogous pattern was observed by Barden-O’Fallon et al. where the utilization of services in private clinics, including vaccination, was most affected by the Ebola crisis [20]. Discrepancies in the results observed between the public and private sectors can be explained by the fact that all public healthcare facilities did not suspend the delivery of its services throughout the COVID-19 pandemic as per the recommendations of national public health authorities while some private physicians limited their services to urgent cases. Moreover, the economic crisis ongoing in the country may have shifted care seeking to primary healthcare centers where vaccines are free of charge. Another justification might be the accessibility to specific private clinics that can be challenging due to license plate restrictions, curfew measures and clinics’ locations, whereas primary healthcare centers are often available in all areas.

When it comes to results on the provision of vaccines from the national immunization calendar, OPV and measles vaccines have witnessed the highest reductions in both the public and private sectors. Besides the CDC report mentioned earlier, there are currently no studies in the literature assessing the changes in the utilization of specific vaccines during the COVID-19 pandemic. Nevertheless, decrease rates for measles vaccine detected in our study (-53.3% in the private sector and -38% in the public sector) were greater than those stated in a report from the Ebola epidemic indicating a 20% decrease in measles coverage during December 2014 as compared to the previous year [22]. The inconsistencies with the literature can be associated with the difference in containment measures between the epidemics and length of the assessment period. When it comes to hepatitis A vaccine, which is administered solely in the private sector however deemed essential and recommended as per the Lebanese calendar developed by the Lebanese Pediatric Society, utilization rates has been reduced to more than half which indicates that the provision of vaccines that are excluded from the national immunization calendar are equally affected.

The main strength of our study is first its originality since it is the only study to assess the impact of COVID-19 pandemic on the utilization of RI services in Lebanon and the MENA region. In addition, vaccination data were collected from all sectors operating in the immunization field in Lebanon, hence rendering the results more reflective of the global vaccine usage in the country. A methodological strength point worth mentioning is the adjustment for the distribution of routine vaccination services between the public and private sectors when calculating the national decrease rate in the utilization of immunization services which further increases the accuracy of the reported rate.

Conversely, several limitations that merits discussion were detected in our study. To begin with, vaccine usage was assessed based on vaccine administration which is a proxy-indicator of vaccination coverage, rather than the latter itself. This was accomplished for ease of access to information and due to the impracticality of conducting coverage studies in pandemic circumstances. Moreover, assessing the utilization of vaccination services in the private sector was associated with several limitations. First, a relatively low survey response rate (26%) was identified, questioning the representativeness of the sample. Another limitation might be related to the methodology of our study where decrease rates in vaccine usage were determined by pediatricians based on subjective estimations as computerized patient records are rarely available. Hence, an overestimation of the observed rates is conceivable, and both recall and information biases are introduced. On the other hand, vaccination figures from the public sector are deemed of greater objectivity since they were extracted from electronic reports communicated on a regular basis by public immunization facilities with the ministry of public health.

## Conclusion

In summary, our results suggest a substantial decrease in the utilization of RI services in both the public and private sectors as a result of the COVID-19 pandemic. Among vaccines of the national immunization calendar, the provision of OPV and measles vaccines was mostly affected during the studied period in both sectors, whereas hepatitis A vaccine witnessed the most significant decrease in usage among vaccines administered only in the private sector.

Our study findings highlight the need for various stakeholders in the immunization field to implement interventions aiming at enhancing RI in all sectors, as well as raising awareness among the general public and healthcare providers on the importance of sustaining immunization services’ provision during the COVID-19 pandemic especially with the potential burden associated with occurrence of outbreaks of VPDs on the economy in a country struggling to overcome its financial crisis.

The evidence generated through this research can be the milestone for projecting the occurrence of future outbreaks of VPDs in Lebanon, thus increasing the preparedness levels of public health authorities to prevent and respond to any potential infectious diseases’ crisis.

## Supporting information

S1 File Data collection tool: English version. (DOCX)

S2 File De-identified dataset. (XLSX)

## References

[1] UNICEF. Over 13 million children did not receive any vaccines at all even before COVID-19 disrupted global immunization. 2020 April 24 [Cited 2020 June 1]. https://www.unicef.org/press-releases/over-13-million-children-did-not-receive-any-vaccines-all-even-covid-19-disrupted

[2] UNICEF. Statement by UNICEF Executive Director Henrietta Fore on the Disruption of Immunization and Basic Health Services due to the COVID-19 Pandemic. 2020 March 25 [Cited 2020 April 15]. https://www.unicef.org/press-releases/statement-unicef-executive-director-henrietta-fore-disruption-immunization-and-basic

[3] Gavi, The Vaccine Alliance. Can routine immunisation be carried out safely during the covid-19 pandemic? 2020 April 15 [Cited 2020 April 10]. https://www.gavi.org/vaccineswork/can-routine-immunisation-be-carried-out-safely-during-covid-19-pandemic

[4] Al Jazeera. Measles: In Ebola’s shadow, a quiet killer is on a rampage in DRC. 2020 April 7 [Cited 2020 April 10]. https://www.aljazeera.com/news/2020/04/measles-ebola-shadow-quiet-killer-rampage-drc-200406164502568.html

[5] Suk JE, Jimenez AP, Kourouma M, Derrough T, Baldé M, Honomou P, et al (2016). Post-Ebola measles outbreak in Lola, Guinea, January–June 2015. J Emerg Infect Dis. 2016; 22(6):1106 10.3201/eid2206.151652 27191621 PMC4880080

[6] UNICEF. Children in the Democratic Republic of the Congo at risk from killer measles, cholera epidemics. 2020 March 31 [Cited 2020 April 15]. https://www.unicef.org/press-releases/children-democratic-republic-congo-risk-killer-measles-cholera-epidemics

[7] Craig AT, Heywood AE, & Worth H (2020). Measles epidemic in Samoa and other Pacific islands. Lancet Infect Dis. 2020; 20(3): 273–275. 10.1016/S1473-3099(20)30053-0 32112752

[8] Bachmaha, M. Vaccination Crisis in Ukraine: Its Origins and Consequences. Krytyka, Thinking Ukraine. 2016 October [Cited 2020 April 10]. https://krytyka.com/en/ukraines-public-health-challenge/articles/vaccination-crisis-ukraine-its-origins-and-consequences

[9] WHO. Ukraine: WHO and UNICEF estimates of immunization coverage: 2014 revision. 2014 [Cited 2020 April 22]. https://apps.who.int/immunization_monitoring/globalsummary/countries?countrycriteria[country][]=UKR

[10] WHO. Ukraine restores immunization coverage in momentous effort to stop measles outbreak that has affected more than 12 000 this year. 2018 May 4 [Cited 2020 April 13]. http://www.euro.who.int/en/countries/ukraine/news/news/2018/05/ukraine-restores-immunization-coverage-in-momentous-effort-to-stop-measles-outbreak-that-has-affected-more-than-12-000-this-year

[11] Bagcchi S. Inadequate vaccine coverage fuels polio outbreak in Ukraine. Lancet Infect Dis. 2020; 15(11): 1268. 10.1016/S1473-3099(15)00367-9 26531041

[12] WHO. Guiding principles for immunization activities during the COVID-19 pandemic. 2020 March 26 [Cited 2020 April 1]. https://apps.who.int/iris/bitstream/handle/10665/331590/WHO-2019-nCoV-immunization_services-2020.1-eng.pdf

[13] The Ministry of Public Health. COVID 19 Operational Plan-Lebanon. 2020 March 10. https://www.moph.gov.lb/userfiles/files/News/COvid%20operation%20plan-converted.pdf

[14] The Ministry of Public Health. MoPH epidemiological surveillance unit. Measles weekly bulletin. 2019.

[15] WHO. WHO vaccine-preventable diseases: monitoring system. 2020 global summary. 2020 [Internet]. https://apps.who.int/immunization_monitoring/globalsummary/estimates?c=LBN

[16] MoPH. National Calendar for Vaccination. Ministry of Public Health. 2016 [Internet]. https://www.moph.gov.lb/userfiles/files/HealthCareSystem/EPI/NationalCalendarforVaccination.pdf

[17] WHO, MoPH. Expanded Programme on Immunization District-Based Immunization Coverage Cluster Survey. World Health Organization. 2018 May 30 [Report]. https://reliefweb.int/sites/reliefweb.int/files/resources/63859.pdf

[18] McDonald HI, Tessier E, White JM, Woodruff M, Knowles C, Bates C, et al Early impact of the coronavirus disease (COVID-19) pandemic and physical distancing measures on routine childhood vaccinations in England, January to April 2020. Euro Surveill. 2020 5;25(19):2000848 10.2807/1560-7917.ES.2020.25.19.2000848 32431288 PMC7238742

[19] Santoli JM. Effects of the COVID-19 pandemic on routine pediatric vaccine ordering and administration—United States, 2020. MMWR. 2020; 69 10.15585/mmwr.mm6919e2 32407298

[20] Barden-O’Fallon J, Barry MA, Brodish P, & Hazerjian J. Rapid assessment of Ebola-related implications for reproductive, maternal, newborn and child health service delivery and utilization in Guinea. PLoS Curr. 2015; 7 10.1371/currents.outbreaks.0b0ba06009dd091bc39ddb3c6d7b0826 26331094 PMC4542265

[21] UNFPA. Rapid Assessment of Ebola Impact on Reproductive Health Services and Service Seeking Behaviour in Sierra Leone. 2015 March [Internet]. https://reliefweb.int/sites/reliefweb.int/files/resources/UNFPA%20study%20_synthesis_March%2025_final.pdf

[22] Assessment Capacities Project (ACAPS). Ebola outbreak in West Africa impact on health service utilisation in Sierra Leone. 2015 March 25 [Internet]. https://www.acaps.org/sites/acaps/files/products/files/f_impact_on_health_service_utilisation_in_sierra_leone_march_2015.pdf

