# Supplementary material for: Impact of COVID-19 pandemic on the utilization of routine immunization services in Lebanon
Source: PLoS One. 2021 Feb 17;16(2):e0246951. doi: 10.1371/journal.pone.0246951 (PMC7888608; doi:10.1371/journal.pone.0246951)
Supplement: S1 File — (DOCX) [file pone.0246951.s001.docx]

*Dear Doctor,*

*The country wide political unrest due to the October 17 uprising followed by the Covid-19 pandemic and the implementation of social distancing measures in Lebanon resulted in alterations in the population's health seeking behaviors, of which is the utilization of routine immunization services.*

*Connecting Research to Development (CRD), a Lebanese research firm, is conducting a study aiming to assess the gap in routine immunization activities in the public and private sectors as a result of the Covid-19 pandemic in order to project the occurrence of potential outbreaks of vaccine preventable diseases after the subsidence of the pandemic, in collaboration with Dr. Abdulrahman Bizri, chairman of the National Certification Committee (NCC) and Dr. Bernard Gerbaka, vice chairman of the EPI technical Committee. The study is done in the aim of informing the national health authorities.*

*For that, we are kindly asking you to answer few questions aiming to evaluate the routine immunization in the private sector. The time needed to fill the survey is around 5 minutes. Anonymity and confidentiality of the survey are completely preserved.*

*Your cooperation is highly appreciated.*

| **#** | **QUESTION** | **CODING CATEGORIES** | | **SKIP** |
| --- | --- | --- | --- | --- |
| **1** | Do you provide routine immunization services? | Yes  No  I prefer not to answer | 1  2  97 | ⇨END  ⇨END |
| **2** | Kindly indicate the geographical area(s) where you practice. | Akkar  North  Mount Lebanon  Beirut  South  Bekaa  Nabatiyeh  Baalback-Hermel  I prefer not to answer | 1  2  3  4  5  6  7  8  97 |  |

| **A** | **Routine Immunization between October 2019 and April 2020** |
| --- | --- |

| **#** | **QUESTION** | **CODING CATEGORIES** | | **SKIP** |
| --- | --- | --- | --- | --- |
| **3** | Have you witnessed any decrease in routine immunization services utilization between October 2019 and April 2020? | Yes  No  I do not know  I prefer not to answer | 1  2  98  97 | ⇨END  ⇨END  ⇨END |
| **4** | Kindly indicate an estimation of the overall decrease rate in routine immunization services during that period (*in percent*) as compared to previous years. | Decrease rate: ___ ___ %  I do not know  I prefer not to answer | 98  97 |  |

| **B** | **Utilization of Specific Vaccines between October 2019 and April 2020** |
| --- | --- |

| **5** | Have you witnessed any decrease in the number of **OPV** vaccines you are giving in your clinic between October 2019 and April 2020 compared to previous years? | Yes  No  I do not know  I prefer not to answer | 1  2  98  97 | ⇨7  ⇨7  ⇨7 |
| --- | --- | --- | --- | --- |
| **6** | Kindly indicate an estimation of the decrease rate in the number of OPV vaccines you are giving in your clinic during that period (in percent) as compared to previous years. | Decrease rate: ___ ___ %  I do not know  I prefer not to answer | 98  97 |  |
| **7** | Have you witnessed any decrease in the number of **IPV-containing vaccines** you are giving in your clinic between October 2019 and April 2020 compared to previous years? | Yes  No  I do not know  I prefer not to answer | 1  2  98  97 | ⇨9  ⇨9  ⇨9 |
| **8** | Kindly indicate an estimation of the decrease rate in the number of IPV-containing vaccines you are giving in your clinic during that period (in percent) as compared to previous years. | Decrease rate: ___ ___ %  I do not know  I prefer not to answer | 98  97 |  |
| **9** | Have you witnessed any decrease in the number of **measles** vaccines you are giving in your clinic between October 2019 and April 2020 compared to previous years? | Yes  No  I do not know  I prefer not to answer | 1  2  98  97 | ⇨11  ⇨11  ⇨11 |
| **10** | Kindly indicate an estimation of the decrease rate in the number of measles vaccines you are giving in your clinic during that period (in percent) as compared to previous years. | Decrease rate: ___ ___ %  I do not know  I prefer not to answer | 98  97 |  |
| **11** | Have you witnessed any decrease in the number of **DTP-containing vaccines** you are giving in your clinic between October 2019 and April 2020 compared to previous years? | Yes  No  I do not know  I prefer not to answer | 1  2  98  97 | ⇨13  ⇨13  ⇨13 |
| **12** | Kindly indicate an estimation of the decrease rate in the number of DTP-containing vaccines you are giving in your clinic during that period (in percent) as compared to previous years. | Decrease rate: ___ ___ %  I do not know  I prefer not to answer | 98  97 |  |
| **13** | Have you witnessed any decrease in the number of **Hepatitis B or HepB-containing vaccines** you are giving in your clinic between October 2019 and April 2020 compared to previous years? | Yes  No  I do not know  I prefer not to answer | 1  2  98  97 | ⇨15  ⇨15  ⇨15 |
| **14** | Kindly indicate an estimation of the decrease rate in the number of Hepatitis B or HepB-containing vaccines you are giving in your clinic during that period (in percent) as compared to previous years. | Decrease rate: ___ ___ %  I do not know  I prefer not to answer | 98  97 |  |
| **15** | Have you witnessed any decrease in the number of **measles/mumps/rubella (MMR)** vaccines you are giving in your clinic between October 2019 and April 2020 compared to previous years? | Yes  No  I do not know  I prefer not to answer | 1  2  98  97 | ⇨17  ⇨17  ⇨17 |
| **16** | Kindly indicate an estimation of the decrease rate in the number of MMR vaccine you are giving in your clinic during that period (in percent) as compared to previous years. | Decrease rate: ___ ___ %  I do not know  I prefer not to answer | 98  97 |  |
| **17** | Have you witnessed any decrease in the number of **PCV** vaccines you are giving in your clinic between October 2019 and April 2020 compared to previous years? | Yes  No  I do not know  I prefer not to answer | 1  2  98  97 | ⇨19  ⇨19  ⇨19 |
| **18** | Kindly indicate an estimation of the decrease rate in the number of PCV vaccines you are giving in your clinic during that period (in percent) as compared to previous years. | Decrease rate: ___ ___ %  I do not know  I prefer not to answer | 98  97 |  |
| **19** | Have you witnessed any decrease in the number of **Hepatitis A** vaccines you are giving in your clinic between October 2019 and April 2020 compared to previous years? | Yes  No  I do not know  I prefer not to answer | 1  2  98  97 | ⇨21  ⇨21  ⇨21 |
| **20** | Kindly indicate an estimation of the decrease rate in the number of Hepatitis A vaccines you are giving in your clinic during that period (in percent) as compared to previous years. | Decrease rate: ___ ___ %  I do not know  I prefer not to answer | 98  97 |  |
| **21** | Which period had the most significant drop in routine vaccination? | October-November 2019  December 2019-January 2020  February-April 2020 (confinement period)  I do not know  I prefer not to answer | 1  2  3  98  97 |  |
